# nDNA-prot: identification of DNA-binding proteins based on unbalanced classification

**DOI:** 10.1186/1471-2105-15-298

**Published:** 2014-09-08

**Authors:** Li Song, Dapeng Li, Xiangxiang Zeng, Yunfeng Wu, Li Guo, Quan Zou

**Affiliations:** School of Information Science and Technology, Xiamen University, Xiamen, Fujian 361005 China; Department of Internal Medicine-Oncology, The Fourth Hospital in Qinhuangdao, Qinhuangdao, Hebei 066000 China; Department of Epidemiology and Biostatistics and Ministry of Education Key Lab for Modern Toxicology, School of Public Health, Nanjing Medical University, Nanjing, Jiangsu 210029 China

**Keywords:** DNA-binding protein, Ensemble classifier, Unbalanced dataset, Bioinformatics

## Abstract

**Background:**

DNA-binding proteins are vital for the study of cellular processes. In recent genome engineering studies, the identification of proteins with certain functions has become increasingly important and needs to be performed rapidly and efficiently. In previous years, several approaches have been developed to improve the identification of DNA-binding proteins. However, the currently available resources are insufficient to accurately identify these proteins. Because of this, the previous research has been limited by the relatively unbalanced accuracy rate and the low identification success of the current methods.

**Results:**

In this paper, we explored the practicality of modelling DNA binding identification and simultaneously employed an ensemble classifier, and a new predictor (nDNA-Prot) was designed. The presented framework is comprised of two stages: a 188-dimension feature extraction method to obtain the protein structure and an ensemble classifier designated as imDC. Experiments using different datasets showed that our method is more successful than the traditional methods in identifying DNA-binding proteins. The identification was conducted using a feature that selected the minimum Redundancy and Maximum Relevance (mRMR). An accuracy rate of 95.80% and an Area Under the Curve (AUC) value of 0.986 were obtained in a cross validation. A test dataset was tested in our method and resulted in an 86% accuracy, versus a 76% using iDNA-Prot and a 68% accuracy using DNA-Prot.

**Conclusions:**

Our method can help to accurately identify DNA-binding proteins, and the web server is accessible at http://datamining.xmu.edu.cn/~songli/nDNA. In addition, we also predicted possible DNA-binding protein sequences in all of the sequences from the UniProtKB/Swiss-Prot database.

**Electronic supplementary material:**

The online version of this article (doi:10.1186/1471-2105-15-298) contains supplementary material, which is available to authorized users.

## Background

A DNA-binding protein is a type of composite protein that is comprised of a combination of structural proteins and is found in the chromosomes and DNA. These proteins perform an important role in the combination and separation of single-stranded DNA and in the detection of DNA damage. Other functions of DNA-binding proteins include stimulation of the nuclease, helicase and strand exchange proteins; transcription at the initiation site; and protein-protein interactions. DNA-binding proteins have important functions in the biological field. Currently, an increasing number of researchers are attempting to identify DNA-binding proteins from other multifarious proteins, and the number of proteins being extracted is rapidly increasing. In 2011, the number of protein sequences in the Swiss-Prot database [1] was more than 100-times greater than in 1986 [2]. Unfortunately, extremely unbalanced data has caused multiple drawbacks in the recent methods for the identification of DNA-binding proteins. Because of this, a quick and effective approach for the identification of DNA-binding proteins is required.

In recent years, an increasing number of feature extractions has been tested in the field of machine learning and biology. Lin and Zou et al. [3] used a 188-dimensional (188D) feature extraction method, which was performed by considering the constitution, physicochemical properties, and distribution of the amino acids [4]. A physicochemical distance transformation (PDT) approach, which is related to the physicochemical properties of amino acids, [5] has also been proposed. In the 188D method, the first 20 feature vectors are obtained based on the probability that every amino acid appears in a given protein sequence. Based on the protein’s physicochemical properties, the remaining 160 feature vectors can then be realised. Patel et al. [6] improved the sequence similarity matrices and used an artificial neural network (ANN), which is a standard back-propagation training algorithm for a feed-forward neural network. Among 1,000 proteins, which included only 62 sequence features, a total accuracy of 72.99% was obtained. Analogously, Cheng et al. [7] also proposed a recurrent neural network that was designed to solve the non-smooth convex optimisation problem. Bhardwaj et al. [8] studied the DNA-binding residues that appear on the protein surface using the residue features that differentiate DNA-binding proteins from non-DNA-binding proteins, and a management alternative was applied as a follow-up to improve the prediction results. Studies have also demonstrated some of the available feature extraction means [9]. According to the protein position-specific scoring matrix, Zou et al. [3] extracted a 20D feature from protein sequences, and in 1992, Brown et al. [10] proposed the n-gram natural language algorithm. This type of algorithm, also applied in another previous study [11], obtains the feature vectors by using a probability calculation. The Basic Local Alignment Search Tool (BLAST), which is based on a position-specific scoring matrix, has also been applied to detect remote protein homology [12].

The abovementioned approaches have all been used to distinguish DNA-binding proteins from non-DNA-binding proteins. In 1999, Nordhoff et al. [11] described the use of mass spectrometry to identify DNA-binding. Gao et al. [13] developed a method based on a knowledge-based method (i.e., DNA-binding domain hunter) and demonstrated how to deduce DNA-binding protein remnants according to the corresponding templates. Loris et al. [12], via a genetic algorithm, discussed the combination of feature extraction approaches with a group of amino acid alphabets. Langlois et al. [14] compared BLAST with a standard sequence alignment technique and discussed the method by which general mechanisms were captured by concrete rules. In 2011, Lin et al. [2], using the grey model, introduced a method for differentiating large-scale DNA-binding proteins by analysing the modality of the pseudo amino acid constitution. Many approaches have also been used to categorise the experimental data in the bioinformatics field. The abovementioned methods can be categorised as follows: Random Forest (RF) [14–17], Support Vector Machine (SVM) [9, 18–22], Dynamic selection and Circulating Combination-based ensemble Clustering (LibD3C) [23, 24], ANN [25–29], k-nearest neighbours (KNN) algorithm [30], and bagging [31].

The founding recognition rate of DNA-binding proteins has also been obtained, at a lower accuracy, using the existing methods rather than by using methods from the other two categories. Additionally, DNA-binding protein classification is an unresolved issue because the results of previous research on the introduction of a number-based sampling strategy showed a high false-positive rate in the extended dataset. As a result, new DNA-binding proteins were not identified. Ahmad and Sarai [27] demonstrated that using the charge and moment information under a hybrid predictor condition resulted in an 83.9% accuracy via a cross validation. The quadrupole moment, using single-variable predictors, resulted in a 73.7% accuracy. Qian et al. [32] verified the association between the DNA-binding preference and the endogenous transcription factors and reached an accuracy rate of 76.6% when using the Jackknife cross-validation test as a predictor. All of these results have exhibited disadvantages though [2]. For example, only some predictors are available on websites where their functions are demonstrated. Thus, an insufficient amount of data contributes to the difficulty in analysing and comparing the results. Currently, the results of many previous studies have not been authenticated, thus impeding the research and development of bioinformatics to some extent. Therefore, an enhanced accuracy rate is a significant research goal.

In light of the current problems, we developed a predictor that addresses the drawbacks of the previously developed predictors. We conducted a series of experiments following a preparation process involving a general selection in addition to data processing. All of the training datasets were obtained from the Universal Protein (UniProt) KB/Swiss-Prot database, which provides high-quality and comprehensive protein sequence resources. We developed a complete dataset that includes an integrated negative-sample dataset. Subsequently, we determined a suitable feature extraction method to reinforce the predictor. We chose the 188D feature extraction method, which is based on the physicochemical properties of proteins. Due to the unsatisfactory performance of the current single classifiers, we applied an ensemble classification prediction algorithm designated as “imDC” to our classification. imDC is based on an unbalanced data research and machine-learning algorithm. The determination of a cross-validation approach to inspect the test dataset was the next important step in the process. Inappropriate cross-validation methods may lead to a deviation in the results and the subsequent failure of the predictor. Finally, a user-friendly web-server that effectively discerns the DNA-binding proteins was developed for checking and verification and for further academic exchanges. Detailed descriptions are provided in the Methods Section.

## Methods

### Pre-processing work

We selected our DNA-binding protein sequences from the website http://www.uniprot.org/ and obtained the data from the UniProtKB/Swiss-Prot database. We used the keyword “DNA-binding” to search for and select the datasets. More than 3,000,000 protein sequences were obtained initially, so we reduced the number of sequences by adding restrictions. The number of protein sequences was reduced to 44,996 when we added restrictions, that is “sequence length from 50 to 6,000” and “reviewed: yes.” Protein sequences with lengths less than 50 amino acids may be incomplete, but those with lengths greater than 6,000 amino acids may be too complex. The sequences that were obtained using the abovementioned limits comprised the initial positive dataset. Twenty types of amino acids and six letters (*b*, *j*, *o*, *u*, *x*, and *z*) were removed. The data downloaded from the database are normally very similar, and such similarities could affect our experimental results. Therefore, we removed any redundant data using cluster database–high identity with tolerance (CD–HIT) [33] with a threshold of 40%. Currently, our positive dataset has 9,676 protein sequences. Every sequence belongs to one or two protein families (PFAMs) [34], and similar sequences belong to the same family. We identified all of the PFAMs from the positive datasets and deleted the redundant PFAMs. We extracted the longest sequences from every PFAM and entered them in the positive dataset, which contained 1,353 protein sequences. We created a file named “PF_all” and deleted the PFAMs where the positive datasets belonged. We also obtained a negative dataset that contained 9,361 protein sequences.

### Feature extraction

Zou et al. [3] analysed the hierarchical classification of protein folds by using the 188D feature extraction method. The 188D feature extraction method, proposed by Cai et al. [19] in 2003, is based on protein physicochemical properties. In this study, the 188D features were constructed in four different ways.

**Figure 1:**
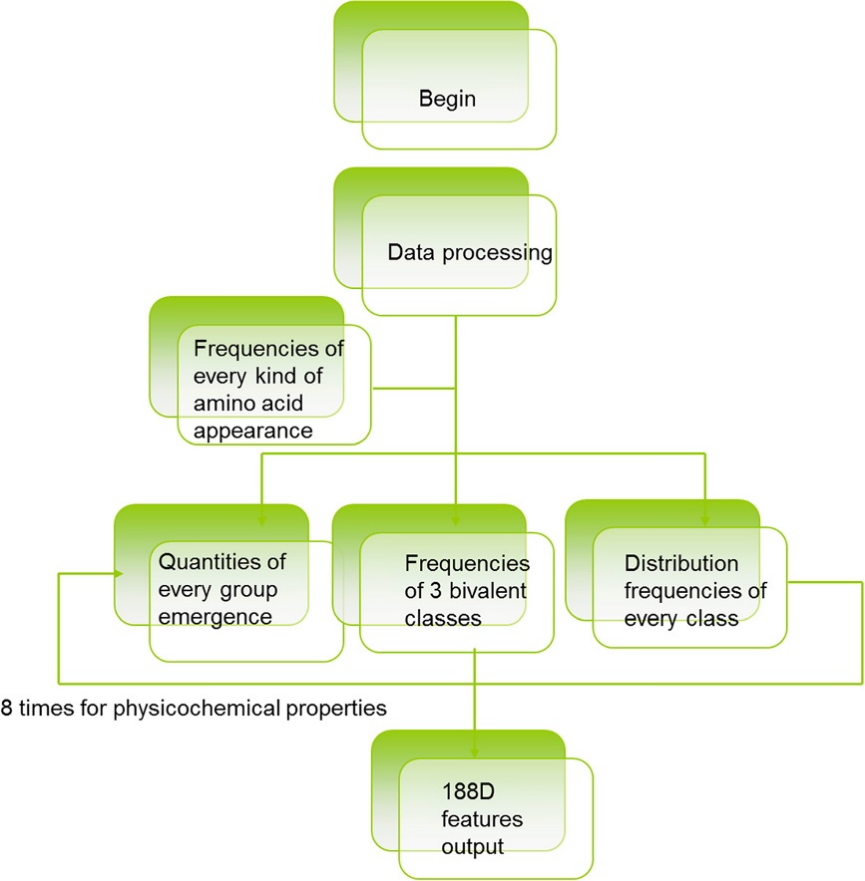
**Flow construction of the 188D feature extraction method.** For the loop body, the number of physicochemical properties is equivalent to the number of loops.

The first 20 dimension features were obtained by calculating the appearance frequency of every amino acid. Subsequently, the amino acids were divided into three different categories based on the protein’s properties. For example, when based on the surface tension of the protein, the amino acids were grouped into GQDNAHR, CPNVEQIL, and KMHFRYW. The quantities of each group emergence became the next three dimension features. The frequencies of three bivalent classes, which were shown in the original sequence, acted as the next three dimension features. Dividing the entire protein sequence into five equal parts and calculating the distribution frequencies of each class in the five parts resulted in the final fifteen dimension features. Therefore, for every protein property, 21 dimension features existed, and eight types of physicochemical properties were used in the 188D analysis. Consequently, 168 dimension features were used as part of the features. A straightforward composition graph of the features is illustrated in Figure 1.

### Classifier selection

In the field of machine learning, which has a relatively mature development, single classifiers have gradually begun to show drawbacks. In the traditional machine-learning algorithm, the data are mostly sacrificed and the quantity of classifier numbers is inferior. The minority samples are inevitably ignored, and a high false-positive rate is obtained. The main solution to the problem is comprised of two methods: data and algorithm aspects. Currently, the majority of the protein sequences in the field of bioinformatics are extremely unbalanced. To use the minority samples efficiently and to avoid the associated lack of data and information, we propose an improved algorithm based on ensemble learning.

**Figure 2:**
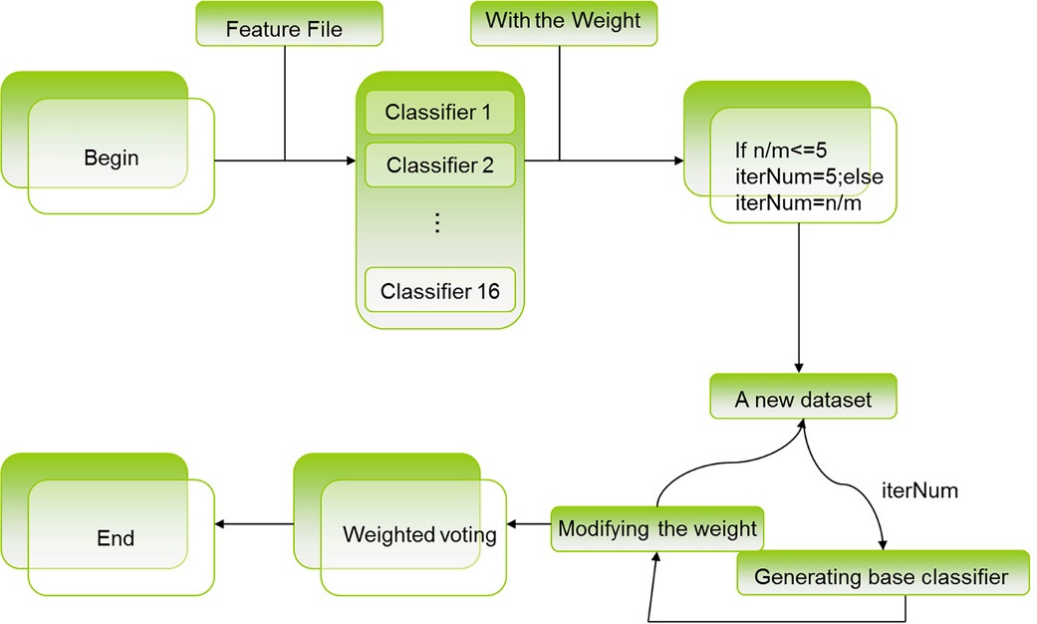
**Framework of the ensemble classifier imDC.** *n* represents the number of minority samples, and *m* stands for the number of majority samples. The loop body is run for iterNum times.

In 1995, Krogh et al. [35] proposed that a large difference in base classifiers leads to a high classification effect after the ensemble. Therefore, in the ensemble classifier, which includes several classifiers, we adopted 16 common sorting algorithms such as LibSVM, NativeBayes, Sequential Minimal Optimization (SMO), IBk, RF, and J48. Five types of high-quality sorting algorithms were selected to circuit train the cycle of new formation training datasets. Subsequently, the ensemble classifier was used to apply a weighted vote to the prediction results of the base classifiers, and the final classification results were obtained. The algorithm of the flow diagram is shown in Figure 2.

### Experiments

#### Data

UniProt is a database that supplies the bioinformatics field with comprehensive, superior-quality results; users can freely access protein sequences and information on protein functions. We obtained the majority of our datasets from Swiss-Prot in the UniProt Knowledgebase, which contains 519,348 note entries in the version released in August 2010. We selected 44,996 protein sequences for our initial dataset. Many similar amino acid compositions or analogous protein functions and structures in the protein sequences were present in the datasets downloaded from the website. Similar amino acid compositions are referred to as having “sequence identity”, which describes the same proteins or nucleic acids that occur at the same position in two sequences.

CD–HIT, designed by Lin in 2006, is a tool for removing redundant sequences [33]. According to this method, all of the sequences are placed in order according to sequence length. The order then comprises a sequence class starting from the longest sequence, and all of the sequences are disposed of, one by one. A representative sequence exists in every sequence class. A new sequence joins the sequence class if the new sequence is similar to the representative sequence of the class. If no similarity exists, a new sequence class is developed. This method is very rapid because a comparison is not required if the same word length between a new sequence and the original sequence does not satisfy the comparative principle. Moreover, the use of an index table also expedites the computational pace. When deleting the redundant sequences in CD–HIT, we chose a series of thresholds, including the range from 0.4 to 0.9, using intervals of 0.1. By using CD–HIT processing, we obtained a series of results (see Table 1) from our unbalanced dataset. DSet represents the dataset with a threshold of 0.4; DSet1 represents the dataset with a threshold of 0.5, and so forth. DSet was smaller than DSet5 by a total of 156 sequences, showing that DSet has little redundancy. Therefore, we used DSet in the majority of the subsequent experiments.

**Table 1:** **The original dataset and the datasets following the threshold removal**

	DNA-binding	Non DNA-binding	Total
**Original dataset**	1,353	9,361	10,714
**DSet**	1,216	8,536	9,752
**DSet1**	1,219	8,611	9,830
**DSet2**	1,220	8,653	9,873
**DSet3**	1,221	8,670	9,891
**DSet4**	1,221	8,676	9,897
**DSet5**	1,223	8,685	9,908

### Feature analysis and classification performance

In this subsection, we evaluate the classification and choice of feature information. We selected a series of specific metrics to measure the results from our method and those from other existing methods. Because the accuracy rate provides a satisfactory description of the results, it is a good measure for identifying a dataset and showing the classification status. However, in some datasets, when the dataset is extremely unbalanced, the accuracy value may not properly represent the quality of the classifier. For example, in a dataset with 100 samples, a certain classifier may think that all 100 are negative, when in fact, the dataset contains ten positive samples. Because of its 90% accuracy, one may think that the classifier shows excellent performance, yet it failed to identify the positive samples. Therefore, we still need to develop other auxiliary judgment criteria to identify the positive samples. In this paper, we chose the F-measure and receiver operating characteristic (ROC) as the criteria.

Classifiers map a point in the ROC plain, resulting in a curve that passes through the points (0,0) and (1,1) in different thresholds. The values of the area under the ROC curve (AUC) indicate the classifier quality. F-measure is a comprehensive evaluation index based on the condition that a contradiction is present between the precision and recall. We obtained these values by calling the Waikato Environment for Knowledge Analysis (WEKA). The value of the AUC is shown in the ROC area. Computation of the methods for accuracy and the F-measure are shown in the following formulas:
1234

where *TP* denotes the number of positive samples that are divided correctly; *FP* refers to the false-negative samples; and *TN* and *FN* represent the opposite samples.

### Performance with different parameters

The unbalanced data are a special circumstance in the data processing of machine learning. In some situations, a low number of categories is observed for the positive samples. Therefore, a large request for recognition occurs, and a single classifier cannot meet the requirements for processing. Thus, under this condition, an ensemble classifier is used. In this study, we used the ensemble classifier imDC and all of the data were processed using a 188D feature extraction. The reason we adopted this 188D feature extraction method is explained in the following section.

**Figure 3:**
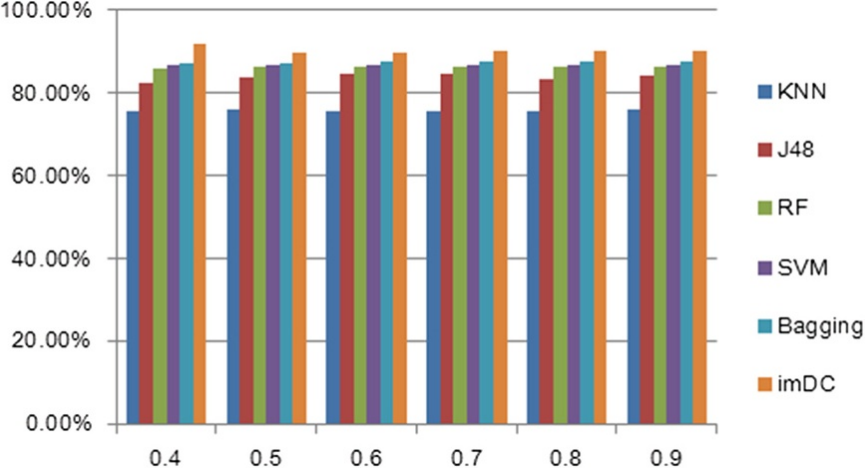
**Comparison of the accuracy between the ensemble classifier imDC and the other classifiers using each of the thresholds.**

In the first experiment, five types of single classifiers (KNN, J48, RF, SVM, and Bagging) were used to examine the performance of the dataset. All of the classifiers underwent a five-fold cross-validation process. As shown in Figure 3, because of the diverse datasets, every classifier showed different results when placed under the different thresholds. As mentioned above, accuracy is the most commonly used evaluation index, and the 0.4 threshold resulted in the best accuracy.

Tables 2 and 3 list the comparison results of the common classifiers and our ensemble classifier in terms of the F-measure and AUC.

**Figure 4:**
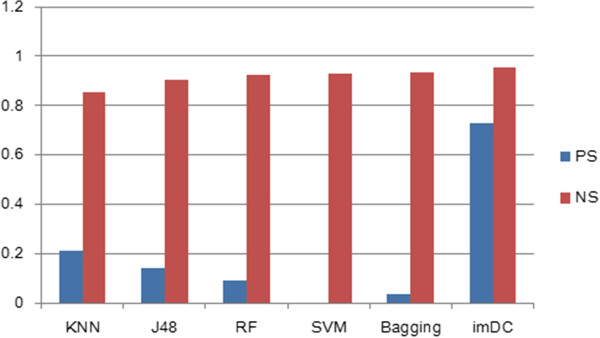
**Comparison of the F**-**measure of the ensemble classifier imDC and the other classifiers using the 0.4 threshold.**

The weighted average of the above results was used. The use of a single classifier can lead to values greater than 0.75, which is acceptable; however, the number of positive samples is not distinguished. In particular, the SVM method was completely unable to identify the positive samples. In Figure 4, we show the drawbacks of using a single classifier in unbalanced datasets.

**Table 2:** **Comparison of the F**-**measure of the ensemble classifier imDC and the other classifiers using each of the thresholds**

	KNN	J48	RF	SVM	Bagging	imDC
**0.4**	0.774	0.808	0.820	0.813	0.820	**0.925**
**0.5**	0.779	0.816	0.823	0.815	0.821	**0.896**
**0.6**	0.775	0.818	0.825	0.815	0.824	**0.892**
**0.7**	0.774	0.819	0.822	0.815	0.825	**0.897**
**0.8**	0.774	0.814	0.823	0.815	0.823	**0.897**
**0.9**	0.779	0.817	0.823	0.815	0.824	**0.896**

**Table 3:** **Comparison of the AUC value of the ensemble classifier imDC and the other classifiers using each of the thresholds**

	KNN	J48	RF	SVM	Bagging	imDC
**0.4**	0.543	0.539	0.624	0.496	0.688	**0.961**
**0.5**	0.544	0.575	0.615	0.496	0.679	**0.931**
**0.6**	0.537	0.585	0.631	0.495	0.690	**0.935**
**0.7**	0.533	0.578	0.621	0.496	0.674	**0.935**
**0.8**	0.533	0.574	0.618	0.496	0.669	**0.934**
**0.9**	0.549	0.579	0.617	0.495	0.679	**0.938**

The other above-normal classifiers failed to show satisfactory results for the positive samples as well. Most of the samples were classified as negative samples, while still indicating a high accuracy. However, a low F-measure, especially under SVM, was observed (PS stands for “positive samples”; NS stands for “negative samples”).

**Figure 5:**
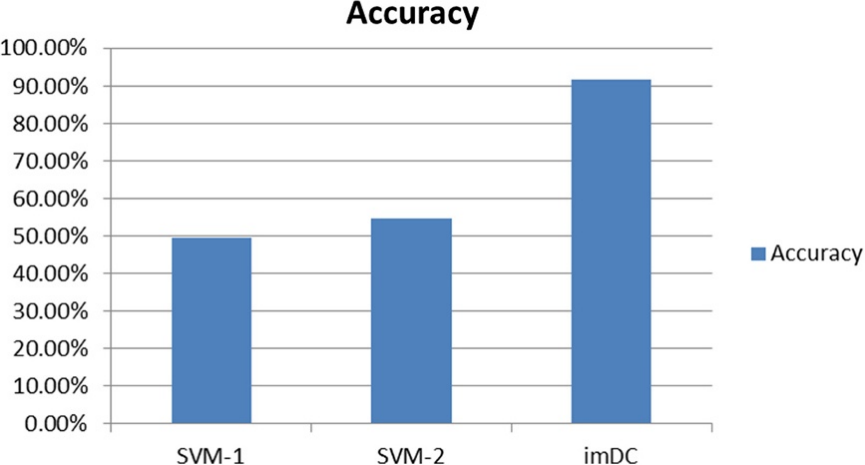
**Comparison between balanced dataset in SVM and unbalanced dataset in imDC.**

Another experiment was carried out to explore the reason of SVM’s low F measure. In this experiment, it included 3 kinds of comparative items. SVM-1 represents dataset with the weight. SVM-2 represents dataset after dimension reduction. Figure 5 shows accuracy results of SVM means under the balanced dataset and imDC under the unbalanced dataset. The y-axis stands for accuracy.

Our tables and figures show only the weighted averages. The specific consequences of the positive and negative cases are not listed. After a series of experiments, a commonly preferred classifier was trained, and all of the comparison results demonstrated that the imbalance classifier, imDC, was very efficient for processing the unbalanced data. Compared with common single classifier, our ensemble classifier has more time loss. However, performance promotion can make up for time loss.

### Performance of the features

In the field of bioinformatics, transforming amino acid sequences into feature values is a critical process, and several methods for this were mentioned in the introduction. Here, we describe the three types of approaches used in the next section in detail.

**Figure 6:**
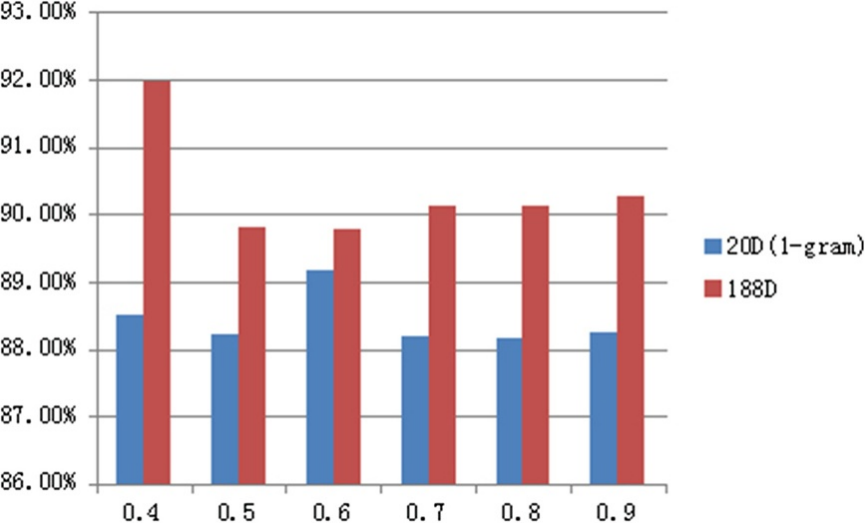
**The accuracy of several feature extraction methods using different thresholds.**

The *n*-gram feature extraction is a method that can easily be understood and implemented. In 1992, Brown et al. [10] demonstrated the *n*-gram models of natural language, which addressed the problem of predicting a word from the previous words in a text sample. Using this approach, we tried to find the relationship between the present and previous amino acids. The n-gram model is based on the assumption that the appearance of the  word is related only to the first *n* - *1* words. Therefore, the probability of the whole sentence is equal to the product of each word’s probability. We obtain these probabilities from the numbers, with which *n* words occur. Calculating the frequency of occurrence of every amino acid in a protein sequence is regarded as an element of a feature vector in 1-gram. Similarly, in 2-gram, the instance of any possible dipeptide occurring will be recorded. Using such a frequency of occurrence to comprise a feature vector, we obtained a total of 420 dimension features. The remainder of the sequences were processed in the same manner. The pseudo amino acid composition (PseAAC or Chou’s PseAAC) and the 188D feature extraction methods showed the same results as the 1-gram method for the first 20D features. PseAAC, proposed by Kuo-Chen Chou in 2001, is also a frequently used feature extraction method to identify certain special proteins. The 188D feature extraction is applied to feature analysis on the basis of these two methods. Cai [1] designed the 188D method in 2003 to predict protein function according to the physicochemical properties of amino acids. For each threshold, the accuracies of the 20D and 188D features were obtained under the conditions of the same imDC classifier (Figure 6). The 20D method data for the three types of methods were similar.

**Figure 7:**
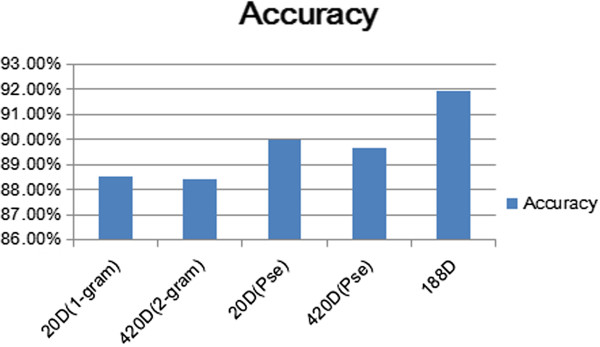
**The accuracy of different datasets using the same ensemble classifier.**

In the subsequent experiments, the same threshold, i.e., 0.4, was used. Figure 7 shows the accuracy results, whose x-axis represents features calculated by *n*-gram, PseAAC, and 188D using the same dataset.

The first 20D features in 188D were exactly the same as the features from the *n*-gram when *n* = 1. It is necessary to determine the contribution rate under this condition. Therefore, we obtained an attribute ranking by using principal component analysis (PCA). The contribution of the first 31 dimension features reached 40.576%. Among the 31 features, only one feature belonged to 1-gram. The PCA results verified that our 188D features were significant.

## Results and discussion

### Data optimisation

**Figure 8:**
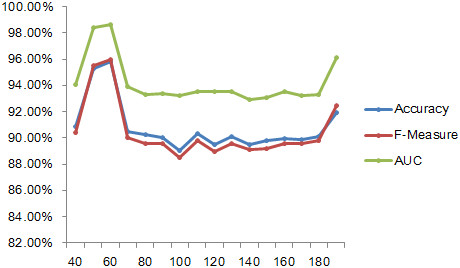
**An indicator variation diagram of the different features after selection.**

Minimum Redundancy Maximum Relevance (mRMR) is a feature selection method used to remove redundancy and to select a compact, effective gene subset from the candidate set [36]. This feature selection method has two forms: mutual information difference (MID) and mutual information quotient (MIQ). In the feature selection, we obtained some of the features from our datasets using MID unified. Based on the existing 188D features, a selection was performed after every 10 features from 40 to 180. The results of the three indicators are shown in Figure 7. The highest consequence was obtained for each indicator when the feature number was 60, among which, sixteen dimension features occurred, which belonged to the first twenty dimensions. More than 70% of the effective features were present among the other 168 dimension features, and the results obtained by our 188D feature extraction approach were significant. The top ten features from the mRMR are listed in Table 4. Figure 8 shows the accuracy, F-measure, and AUC results in terms of the different numbers of features.

**Table 4:** **The rank of features in the mRMR feature selection**

Order	Fea	Name	Score
**1**	23	Fea23	0.017
**2**	41	Fea41	-0.001
**3**	83	Fea83	-0.002
**4**	9	Fea9	-0.002
**5**	36	Fea36	-0.003
**6**	2	Fea2	-0.004
**7**	7	Fea7	-0.003
**8**	155	Fea155	-0.005
**9**	16	Fea16	-0.006
**10**	115	Fea115	-0.005

### Comparison with other software

To verify the model’s performance, we selected, at random, fifty samples as our test dataset, of which ten were positive and forty were negative. Table 5 shows a comparison of the test results obtained with our model compared to the existing web server and software, iDNA-Prot and DNA-Prot. iDNA-Prot was proposed by Lin et al. in 2011, aiming at identifying DNA-binding proteins using random forest with grey model. An available public web-site in Lin et al. makes it comparable. The same reason can also be used in DNA-Prot. The results demonstrate the superiority of our method, both in feature extraction and in ensemble classifiers, especially in the positive sample process.

**Table 5:** **A comparison of the three predictor methods**

	Precision		Accuracy
	Positive	Negative	
**DNA**-**Prot**	20%	80%	68%
**iDNA**-**Prot**	0	95%	76%
**nDNA**-**Prot**	50%	95%	86%

### Further work

There are more than 56 million sequences in the UniProt knowledgebase. We downloaded 545,388 protein sequences that have been reviewed in Swiss-Prot and used our generated model to predict the sequences. A total of 119 protein sequences were identified as DNA-binding proteins. Information about these sequences has been listed in the file Additional file 1. This work will aid in the discovery of more potential DNA-binding proteins.

## Conclusions

The recognition of DNA-binding proteins is rapidly increasing. In this paper, we emphasised the analysis of unbalanced DNA-binding protein data and designed an ensemble classification algorithm to address this imbalance. The presentation of the new ensemble classifier imDC was shown to improve the ease of discriminating DNA-binding proteins from other complex proteins.

After a series of feature extraction comparisons, the 188D feature extraction method suggested the superiority of our unbalanced dataset, even if the improved dimension resulted in the loss of time. Feature selection is necessary to reduce the running time and increase the efficiency of a feature extraction. The feature selection method mRMR efficiently solves this problem. Our paper presents the results of the feature selection, and Table 4 summarises the following points: (1) Amino acids, such as C, H, K, and S, are important in recognising DNA-binding proteins, and (2) the features extracted based on the hydrophobicity contribute to 30% of the top ten features and show the materiality of hydrophobicity. Finding an appropriate feature dimension to achieve the maximum performance of a classifier in all types of thresholds will be considered in the future. In addition, using a simple test to compare our model with the other software, we showed that our method has a greater advantage for processing an unbalanced dataset. A user-friendly recognition prediction system is provided at http://datamining.xmu.edu.cn/~songli/nDNA, where users can submit protein sequences for prediction in a particular format. A quick prediction has already been performed on the DNA-binding protein sequences in the UniProtKB/Swiss-Prot database.

The model built in this paper positively affects the identification of DNA-binding proteins. The results of our research will be adopted in future studies in this field.

## Electronic supplementary material

Additional file 1: **119 Protein Sequences.** (DOC 41 KB)

## References

[1] Boutet E, Lieberherr D, Tognolli M, Schneider M, Bairoch A (2007). Uniprotkb/swiss-prot. Plant Bioinformatics. Humana Press.

[2] Lin W-Z, Fang JA, Xiao X, Chou KC (2011). iDNA-Prot: identification of DNA binding proteins using random forest with grey model. PLoS One.

[3] Lin C, Zou Y, Qin J, Liu X, Jiang Y, Ke C, Zou Q (2013). Hierarchical classification of protein folds using a novel ensemble classifier. PLoS One.

[4] Chen W, Liu X, Huang Y, Jiang Y, Zou Q, Lin C (2012). Improved method for predicting the protein fold pattern with ensemble classifiers. Genet Mol Res.

[5] Liu B, Wang X, Chen Q, Dong Q, Lan X (2012). Using amino acid physicochemical distance transformation for fast protein remote homology detection. PLoS One.

[6] Patel AK, Patel S, Naik PK (2009). Binary classification of uncharacterized proteins into DNA binding/non-DNA binding proteins from sequence derived features using Ann. Dig J Nanomaterials & Biostructures (DJNB).

[7] Cheng L, Hou Z, Lin Y, Tan M, Zhang W, Wu F (2011). Recurrent neural network for non-smooth convex optimization problems with application to the identification of genetic regulatory networks. IEEE Trans Neural Netw.

[8] Bhardwaj N, Lu H (2007). Residue-level prediction of DNA-binding sites and its application on DNA-binding protein predictions. FEBS Lett.

[9] Zou Q, Li X, Jiang Y, Zhao Y, Wang G (2013). BinMemPredict: a web server and software for predicting membrane protein types. Curr Proteomics.

[10] Brown PF, Della Pietra VJ, de Souza PV, Lai JC, Mercer RL (1992). Class-based n-gram models of natural language. Comput Linguist.

[11] Nordhoff E, Krogsdam AM, Jorgensen HF, Kallipolitis BH, Clark BF, Roepstorff P, Kristiansen K (1999). Rapid identification of DNA-binding proteins by mass spectrometry. Nat Biotechnol.

[12] Nanni L, Lumini A (2009). An ensemble of reduced alphabets with protein encoding based on grouped weight for predicting DNA-binding proteins. Amino Acids.

[13] Nimrod G, Schushan M, Szilágyi A, Leslie C, Ben-Tal N (2010). iDBPs: a web server for the identification of DNA binding proteins. Bioinformatics.

[14] Langlois RE, Lu H (2010). Boosting the prediction and understanding of DNA-binding domains from sequence. Nucleic Acids Res.

[15] Ma X, Guo J, Liu HD, Xie JM, Sun X (2012). Sequence-based prediction of DNA-binding residues in proteins with conservation and correlation information. IEEE/ACM Trans Comput Biol Bioinform.

[16] Brown J, Akutsu T (2009). Identification of novel DNA repair proteins via primary sequence, secondary structure, and homology. BMC Bioinformatics.

[17] Fang Y, Guo Y, Feng Y, Li M (2008). Predicting DNA-binding proteins: approached from Chou’s pseudo amino acid composition and other specific sequence features. Amino Acids.

[18] Cai YD, Lin SL (2003). Support vector machines for predicting rRNA-, RNA-, and DNA-binding proteins from amino acid sequence. Biochim et Biophys Acta (BBA)-Proteins and Proteomics.

[19] Cai C, Han L, Ji Z, Chen X, Chen Y (2003). SVM-Prot: web-based support vector machine software for functional classification of a protein from its primary sequence. Nucleic Acids Res.

[20] Kumar M, Gromiha MM, Raghava GP (2007). Identification of DNA-binding proteins using support vector machines and evolutionary profiles. BMC Bioinformatics.

[21] Rashid M, Saha S, Raghava GP (2007). Support Vector Machine-based method for predicting subcellular localization of mycobacterial proteins using evolutionary information and motifs. BMC Bioinformatics.

[22] Liu B, Xu J, Zou Q, Xu R, Wang X, Chen Q (2014). Using distances between Top-n-gram and residue pairs for protein remote homology detection. BMC Bioinformatics.

[23] Zou Q, Wang Z, Wu Y, Liu B, Lin Z, Guan X (2013). An approach for identifying cytokines based on a novel ensemble classifier. BioMed Res Int.

[24] Lin C, Chen W, Qiu C, Wu Y, Krishnan S, Zou Q (2014). LibD3C: ensemble classifiers with a clustering and dynamic selection strategy. Neurocomputing.

[25] Schneider G, Wrede P (1998). Artificial neural networks for computer-based molecular design. Prog Biophys Mol Biol.

[26] Molparia B, Goyal K, Sarkar A, Kumar S, Sundar D (2010). ZiF-Predict: a web tool for predicting DNA-binding specificity in C2H2 zinc finger proteins. Genomics Proteomics Bioinformatics.

[27] Ahmad S, Sarai A (2004). Moment-based prediction of DNA-binding proteins. J Mol Biol.

[28] Keil M, Exner TE, Brickmann J (2004). Pattern recognition strategies for molecular surfaces: III. Binding site prediction with Neural Netw J Comput Chem.

[29] Xu R, Zhou J, Liu B, Yao L, He Y, Zou Q, Wang X (2014). enDNA-Prot: identification of DNA-Binding Proteins by applying ensemble learning. BioMed Res Int.

[30] Cai Y, He J, Li X, Lu L, Yang X, Feng K, Lu W, Kong X (2008). A novel computational approach to predict transcription factor DNA binding preference. J Proteome Res.

[31] Breiman L (1996). Bagging predictors. Machine Learn.

[32] Qian Z, Cai Y-D, Li Y (2006). A novel computational method to predict transcription factor DNA binding preference. Biochem Biophys Res Commun.

[33] Li W, Jaroszewski L, Godzik A (2002). Sequence clustering strategies improve remote homology recognitions while reducing search times. Protein Eng.

[34] Cheng X-Y, Huang WJ, Hu SC, Zhang HL, Wang H, Zhang JX, Lin HH, Chen YZ, Zou Q, Ji ZL (2012). A global characterization and identification of multifunctional enzymes. PLoS One.

[35] Krogh A, Vedelsby J (1995). Neural network ensembles, cross validation, and active learning. Adv Neural Inf Process Syst.

[36] Zhang Y, Ding C, Li T (2008). Gene selection algorithm by combining reliefF and mRMR. BMC Genomics.

