# Supplementary material for: nDNA-prot: identification of DNA-binding proteins based on unbalanced classification
Source: BMC Bioinformatics. 2014 Sep 8;15(1):298. doi: 10.1186/1471-2105-15-298 (PMC4165999; doi:10.1186/1471-2105-15-298)
Supplement: Supplementary file 1 — Additional file 1: 119 Protein Sequences. (DOC 41 KB) [file 12859_2014_6579_MOESM1_ESM.doc]

119 Protein Sequences:

>sp|P29555|ABDA_DROME Homeobox protein abdominal-A OS=Drosophila melanogaster GN=abd-A PE=2 SV=2

>sp|B3LN45|AIM3_YEAS1 Altered inheritance of mitochondria protein 3 OS=Saccharomyces cerevisiae (strain RM11-1a) GN=AIM3 PE=3 SV=1

>sp|C7GUK6|AIM3_YEAS2 Altered inheritance of mitochondria protein 3 OS=Saccharomyces cerevisiae (strain JAY291) GN=AIM3 PE=3 SV=1

>sp|A6ZL53|AIM3_YEAS7 Altered inheritance of mitochondria protein 3 OS=Saccharomyces cerevisiae (strain YJM789) GN=AIM3 PE=3 SV=1

>sp|D3UEK1|AIM3_YEAS8 Altered inheritance of mitochondria protein 3 OS=Saccharomyces cerevisiae (strain Lalvin EC1118 / Prise de mousse) GN=AIM3 PE=3 SV=1

>sp|P38266|AIM3_YEAST Altered inheritance of mitochondria protein 3 OS=Saccharomyces cerevisiae (strain ATCC 204508 / S288c) GN=AIM3 PE=1 SV=3

>sp|Q96L96|ALPK3_HUMAN Alpha-protein kinase 3 OS=Homo sapiens GN=ALPK3 PE=2 SV=2

>sp|Q9VCA8|ANKHM_DROME Ankyrin repeat and KH domain-containing protein mask OS=Drosophila melanogaster GN=mask PE=1 SV=2

>sp|P62287|ASPM_COLGU Abnormal spindle-like microcephaly-associated protein homolog OS=Colobus guereza GN=ASPM PE=3 SV=1

>sp|P62289|ASPM_GORGO Abnormal spindle-like microcephaly-associated protein homolog OS=Gorilla gorilla gorilla GN=ASPM PE=2 SV=1

>sp|Q8IZT6|ASPM_HUMAN Abnormal spindle-like microcephaly-associated protein OS=Homo sapiens GN=ASPM PE=1 SV=2

>sp|P62290|ASPM_HYLLA Abnormal spindle-like microcephaly-associated protein homolog OS=Hylobates lar GN=ASPM PE=3 SV=1

>sp|P62291|ASPM_MACFA Abnormal spindle-like microcephaly-associated protein homolog OS=Macaca fascicularis GN=ASPM PE=2 SV=1

>sp|P62292|ASPM_MACMU Abnormal spindle-like microcephaly-associated protein homolog OS=Macaca mulatta GN=ASPM PE=3 SV=1

>sp|P62293|ASPM_PANTR Abnormal spindle-like microcephaly-associated protein homolog OS=Pan troglodytes GN=ASPM PE=2 SV=1

>sp|P62294|ASPM_PONPY Abnormal spindle-like microcephaly-associated protein homolog OS=Pongo pygmaeus GN=ASPM PE=2 SV=1

>sp|Q9V727|ASX_DROME Polycomb protein Asx OS=Drosophila melanogaster GN=Asx PE=1 SV=1

>sp|Q29A33|ATX2_DROPS Ataxin-2 homolog OS=Drosophila pseudoobscura pseudoobscura GN=Atx2 PE=3 SV=2

>sp|Q8SWR8|ATX2_DROME Ataxin-2 homolog OS=Drosophila melanogaster GN=Atx2 PE=1 SV=1

>sp|P23645|BIB_DROME Neurogenic protein big brain OS=Drosophila melanogaster GN=bib PE=1 SV=2

>sp|Q24266|BTD_DROME Transcription factor btd OS=Drosophila melanogaster GN=btd PE=2 SV=1

>sp|Q24523|BUN2_DROME Protein bunched, class 2/F/G isoform OS=Drosophila melanogaster GN=bun PE=2 SV=4

>sp|Q0DVU4|C3H20_ORYSJ Zinc finger CCCH domain-containing protein 20 OS=Oryza sativa subsp. japonica GN=Os03g0112700 PE=2 SV=1

>sp|Q6PHN1|CCD57_MOUSE Coiled-coil domain-containing protein 57 OS=Mus musculus GN=Ccdc57 PE=2 SV=1

>sp|Q6T8E9|CCNT1_BOVIN Cyclin-T1 OS=Bos taurus GN=CCNT1 PE=1 SV=1

>sp|Q9XT26|CCNT1_HORSE Cyclin-T1 OS=Equus caballus GN=CCNT1 PE=1 SV=1

>sp|O60563|CCNT1_HUMAN Cyclin-T1 OS=Homo sapiens GN=CCNT1 PE=1 SV=1

>sp|Q9QWV9|CCNT1_MOUSE Cyclin-T1 OS=Mus musculus GN=Ccnt1 PE=1 SV=3

>sp|Q8HXN7|CCNT1_PANTR Cyclin-T1 OS=Pan troglodytes GN=CCNT1 PE=2 SV=1

>sp|O61735|CLOCK_DROME Circadian locomoter output cycles protein kaput OS=Drosophila melanogaster GN=Clk PE=1 SV=3

>sp|Q91YB0|CLOCK_NANGA Circadian locomoter output cycles protein kaput OS=Nannospalax galili GN=Clock PE=1 SV=1

>sp|Q9WVS9|CLOCK_RAT Circadian locomoter output cycles protein kaput OS=Rattus norvegicus GN=Clock PE=2 SV=1

>sp|Q5RAK8|CLOCK_PONAB Circadian locomoter output cycles protein kaput OS=Pongo abelii GN=CLOCK PE=2 SV=1

>sp|P41046|CORTO_DROME Centrosomal and chromosomal factor OS=Drosophila melanogaster GN=corto PE=1 SV=2

>sp|P49762|DOA_DROME Serine/threonine-protein kinase Doa OS=Drosophila melanogaster GN=Doa PE=1 SV=2

>sp|P20105|E74EA_DROME Ecdysone-induced protein 74EF isoform A OS=Drosophila melanogaster GN=Eip74EF PE=2 SV=2

>sp|Q7M3M6|E74EF_DROVI Ecdysone-induced protein 74EF OS=Drosophila virilis GN=Eip74EF PE=2 SV=1

>sp|Q4WP03|EAF1_ASPFU Chromatin modification-related protein eaf1 OS=Neosartorya fumigata (strain ATCC MYA-4609 / Af293 / CBS 101355 / FGSC A1100) GN=eaf1 PE=3 SV=1

>sp|Q5B4Q8|EAF1_EMENI Chromatin modification-related protein eaf1 OS=Emericella nidulans (strain FGSC A4 / ATCC 38163 / CBS 112.46 / NRRL 194 / M139) GN=eaf1 PE=3 SV=1

>sp|Q09228|EGL27_CAEEL Egg-laying defective protein 27 OS=Caenorhabditis elegans GN=egl-27 PE=1 SV=2

>sp|O04425|FCA_ARATH Flowering time control protein FCA OS=Arabidopsis thaliana GN=FCA PE=1 SV=2

>sp|O14270|FHL1_SCHPO Fork head transcription factor 1 OS=Schizosaccharomyces pombe (strain 972 / ATCC 24843) GN=fhl1 PE=4 SV=2

>sp|P11088|FILA_MOUSE Filaggrin (Fragment) OS=Mus musculus GN=Flg PE=2 SV=1

>sp|A8MPH9|FOSLD_DROME Transcription factor kayak, isoforms D/sro OS=Drosophila melanogaster GN=kay PE=1 SV=2

>sp|Q9DB00|GON4L_MOUSE GON-4-like protein OS=Mus musculus GN=Gon4l PE=1 SV=3

>sp|Q3T8J9|GON4L_HUMAN GON-4-like protein OS=Homo sapiens GN=GON4L PE=1 SV=1

>sp|Q535K8|GON4L_RAT GON-4-like protein OS=Rattus norvegicus GN=Gon4l PE=2 SV=1

>sp|Q54KX0|GTAN_DICDI GATA zinc finger domain-containing protein 14 OS=Dictyostelium discoideum GN=gtaN PE=4 SV=1

>sp|C0H4W3|HEPF1_PLAF7 Probable ATP-dependent helicase PF08_0048 OS=Plasmodium falciparum (isolate 3D7) GN=PF08_0048 PE=3 SV=1

>sp|Q7KM13|HEY_DROME Hairy/enhancer-of-split related with YRPW motif protein OS=Drosophila melanogaster GN=Hey PE=2 SV=1

>sp|Q6C2N2|HSE1_YARLI Class E vacuolar protein-sorting machinery protein HSE1 OS=Yarrowia lipolytica (strain CLIB 122 / E 150) GN=HSE1 PE=3 SV=1

>sp|Q01778|HUNB_MUSDO Protein hunchback OS=Musca domestica GN=hb PE=3 SV=2

>sp|P16749|ICP27_HCMVA mRNA export factor ICP27 homolog OS=Human cytomegalovirus (strain AD169) GN=UL69 PE=1 SV=1

>sp|Q6SW73|ICP27_HCMVM mRNA export factor ICP27 homolog OS=Human cytomegalovirus (strain Merlin) GN=UL69 PE=3 SV=1

>sp|Q92794|KAT6A_HUMAN Histone acetyltransferase KAT6A OS=Homo sapiens GN=KAT6A PE=1 SV=2

>sp|Q6PDK2|KMT2D_MOUSE Histone-lysine N-methyltransferase 2D OS=Mus musculus GN=Kmt2d PE=1 SV=2

>sp|P10105|LAB_DROME Homeotic protein labial OS=Drosophila melanogaster GN=lab PE=1 SV=2

>sp|Q09260|LAG3_CAEEL Protein lag-3 OS=Caenorhabditis elegans GN=sel-8 PE=1 SV=2

>sp|Q92585|MAML1_HUMAN Mastermind-like protein 1 OS=Homo sapiens GN=MAML1 PE=1 SV=3

>sp|Q6T264|MAML1_MOUSE Mastermind-like protein 1 OS=Mus musculus GN=Maml1 PE=1 SV=2

>sp|P0C6A2|MAMD1_MOUSE Mastermind-like domain-containing protein 1 OS=Mus musculus GN=Mamld1 PE=2 SV=1

>sp|A5D7F6|MAML2_BOVIN Mastermind-like protein 2 OS=Bos taurus GN=MAML2 PE=2 SV=1

>sp|Q8IZL2|MAML2_HUMAN Mastermind-like protein 2 OS=Homo sapiens GN=MAML2 PE=1 SV=2

>sp|Q96JK9|MAML3_HUMAN Mastermind-like protein 3 OS=Homo sapiens GN=MAML3 PE=1 SV=3

>sp|P21519|MAM_DROME Neurogenic protein mastermind OS=Drosophila melanogaster GN=mam PE=2 SV=2

>sp|Q5XI50|MARH7_RAT E3 ubiquitin-protein ligase MARCH7 OS=Rattus norvegicus GN=March7 PE=2 SV=1

>sp|Q9WV66|MARH7_MOUSE E3 ubiquitin-protein ligase MARCH7 OS=Mus musculus GN=March7 PE=2 SV=1

>sp|Q95YM8|MBLK1_APIME Mushroom body large-type Kenyon cell-specific protein 1 OS=Apis mellifera GN=Mblk-1 PE=1 SV=1

>sp|Q96RN5|MED15_HUMAN Mediator of RNA polymerase II transcription subunit 15 OS=Homo sapiens GN=MED15 PE=1 SV=2

>sp|Q21502|MEP1_CAEEL MOG interacting and ectopic P-granules protein 1 OS=Caenorhabditis elegans GN=mep-1 PE=1 SV=2

>sp|Q03825|MSS11_YEAST Transcription activator MSS11 OS=Saccharomyces cerevisiae (strain ATCC 204508 / S288c) GN=MSS11 PE=1 SV=1

>sp|Q54HX6|MYBI_DICDI Myb-like protein I OS=Dictyostelium discoideum GN=mybI PE=3 SV=1

>sp|Q869R9|MYBJ_DICDI Myb-like protein J OS=Dictyostelium discoideum GN=mybJ PE=3 SV=1

>sp|P38996|NAB3_YEAST Nuclear polyadenylated RNA-binding protein 3 OS=Saccharomyces cerevisiae (strain ATCC 204508 / S288c) GN=NAB3 PE=1 SV=1

>sp|Q14686|NCOA6_HUMAN Nuclear receptor coactivator 6 OS=Homo sapiens GN=NCOA6 PE=1 SV=3

>sp|Q9JLI4|NCOA6_RAT Nuclear receptor coactivator 6 (Fragment) OS=Rattus norvegicus GN=Ncoa6 PE=1 SV=2

>sp|Q9JL19|NCOA6_MOUSE Nuclear receptor coactivator 6 OS=Mus musculus GN=Ncoa6 PE=1 SV=1

>sp|P34333|NCOR1_CAEEL Nuclear receptor corepressor 1 OS=Caenorhabditis elegans GN=gei-8 PE=1 SV=4

>sp|Q86NP2|NELFA_DROME Negative elongation factor A OS=Drosophila melanogaster GN=Nelf-A PE=1 SV=2

>sp|O94916|NFAT5_HUMAN Nuclear factor of activated T-cells 5 OS=Homo sapiens GN=NFAT5 PE=1 SV=1

>sp|Q9WV30|NFAT5_MOUSE Nuclear factor of activated T-cells 5 OS=Mus musculus GN=Nfat5 PE=1 SV=2

>sp|D3ZGB1|NFAT5_RAT Nuclear factor of activated T-cells 5 OS=Rattus norvegicus GN=Nfat5 PE=1 SV=1

>sp|Q557I1|NFYC_DICDI Nuclear transcription factor Y subunit gamma OS=Dictyostelium discoideum GN=nfyc-1 PE=3 SV=1

>sp|Q5F2E7|NUFP2_MOUSE Nuclear fragile X mental retardation-interacting protein 2 OS=Mus musculus GN=Nufip2 PE=1 SV=1

>sp|P51521|OVO_DROME Protein ovo OS=Drosophila melanogaster GN=ovo PE=1 SV=2

>sp|P78364|PHC1_HUMAN Polyhomeotic-like protein 1 OS=Homo sapiens GN=PHC1 PE=1 SV=3

>sp|Q8IXK0|PHC2_HUMAN Polyhomeotic-like protein 2 OS=Homo sapiens GN=PHC2 PE=1 SV=1

>sp|Q8CHP6|PHC3_MOUSE Polyhomeotic-like protein 3 OS=Mus musculus GN=Phc3 PE=1 SV=2

>sp|Q8NDX5|PHC3_HUMAN Polyhomeotic-like protein 3 OS=Homo sapiens GN=PHC3 PE=1 SV=1

>sp|P39769|PHP_DROME Polyhomeotic-proximal chromatin protein OS=Drosophila melanogaster GN=ph-p PE=1 SV=2

>sp|P34217|PIN4_YEAST RNA-binding protein PIN4 OS=Saccharomyces cerevisiae (strain ATCC 204508 / S288c) GN=PIN4 PE=1 SV=1

>sp|P78424|PO6F2_HUMAN POU domain, class 6, transcription factor 2 OS=Homo sapiens GN=POU6F2 PE=1 SV=3

>sp|P29617|PROS_DROME Homeobox protein prospero OS=Drosophila melanogaster GN=pros PE=1 SV=3

>sp|Q69IL4|RF2A_ORYSJ Transcription factor RF2a OS=Oryza sativa subsp. japonica GN=RF2a PE=1 SV=1

>sp|P22670|RFX1_HUMAN MHC class II regulatory factor RFX1 OS=Homo sapiens GN=RFX1 PE=1 SV=2

>sp|P48377|RFX1_MOUSE MHC class II regulatory factor RFX1 OS=Mus musculus GN=Rfx1 PE=2 SV=2

>sp|P34758|SCD5_YEAST Protein SCD5 OS=Saccharomyces cerevisiae (strain ATCC 204508 / S288c) GN=SCD5 PE=1 SV=1

>sp|Q8W234|SEUSS_ARATH Transcriptional corepressor SEUSS OS=Arabidopsis thaliana GN=SEU PE=1 SV=1

>sp|P22807|SLOU_DROME Homeobox protein slou OS=Drosophila melanogaster GN=slou PE=2 SV=1

>sp|Q5E9U0|SP2_BOVIN Transcription factor Sp2 OS=Bos taurus GN=SP2 PE=2 SV=1

>sp|Q9D2H6|SP2_MOUSE Transcription factor Sp2 OS=Mus musculus GN=Sp2 PE=2 SV=2

>sp|Q55F37|SRFC_DICDI Transcription factor mef2A OS=Dictyostelium discoideum GN=mef2A PE=2 SV=2

>sp|P52172|SRP_DROME Box A-binding factor OS=Drosophila melanogaster GN=srp PE=1 SV=2

>sp|P11378|STP2_MOUSE Nuclear transition protein 2 OS=Mus musculus GN=Tnp2 PE=2 SV=2

>sp|Q9SR71|TAF12_ARATH Transcription initiation factor TFIID subunit 12 OS=Arabidopsis thaliana GN=TAF12 PE=1 SV=1

>sp|Q64336|TBR1_MOUSE T-box brain protein 1 OS=Mus musculus GN=Tbr1 PE=1 SV=2

>sp|Q9UGU0|TCF20_HUMAN Transcription factor 20 OS=Homo sapiens GN=TCF20 PE=1 SV=3

>sp|Q9EPQ8|TCF20_MOUSE Transcription factor 20 OS=Mus musculus GN=Tcf20 PE=1 SV=3

>sp|O15405|TOX3_HUMAN TOX high mobility group box family member 3 OS=Homo sapiens GN=TOX3 PE=1 SV=2

>sp|Q4P7Q1|VPS27_USTMA Vacuolar protein sorting-associated protein 27 OS=Ustilago maydis (strain 521 / FGSC 9021) GN=VPS27 PE=3 SV=1

>sp|Q5A6T8|WOR3_CANAL White-opaque regulator 3 OS=Candida albicans (strain SC5314 / ATCC MYA-2876) GN=WOR3 PE=1 SV=1

>sp|Q54MP8|Y5837_DICDI Bromodomain and WD repeat-containing DDB_G0285837 OS=Dictyostelium discoideum GN=DDB_G0285837 PE=4 SV=1

>sp|Q8K0L9|ZBT20_MOUSE Zinc finger and BTB domain-containing protein 20 OS=Mus musculus GN=Zbtb20 PE=1 SV=1

>sp|Q15911|ZFHX3_HUMAN Zinc finger homeobox protein 3 OS=Homo sapiens GN=ZFHX3 PE=1 SV=2

>sp|Q61329|ZFHX3_MOUSE Zinc finger homeobox protein 3 OS=Mus musculus GN=Zfhx3 PE=1 SV=1

>sp|B8AX53|ZHD6_ORYSI Zinc-finger homeodomain protein 6 OS=Oryza sativa subsp. indica GN=ZHD6 PE=3 SV=1

>sp|Q688U3|ZHD6_ORYSJ Zinc-finger homeodomain protein 6 OS=Oryza sativa subsp. japonica GN=ZHD6 PE=2 SV=1

>sp|Q8TF68|ZN384_HUMAN Zinc finger protein 384 OS=Homo sapiens GN=ZNF384 PE=1 SV=2

>sp|Q96JM2|ZN462_HUMAN Zinc finger protein 462 OS=Homo sapiens GN=ZNF462 PE=1 SV=3
